# A New pH-Dependent Macrocyclic Rhodamine B-Based Fluorescent Probe for Copper Detection in White Wine

**DOI:** 10.3390/s19204514

**Published:** 2019-10-17

**Authors:** Nour Doumani, Elias Bou-Maroun, Jacqueline Maalouly, Maya Tueni, Adrien Dubois, Claire Bernhard, Franck Denat, Philippe Cayot, Nicolas Sok

**Affiliations:** 1 UMR PAM Procédés Alimentaires et Microbiologiques, Université de Bourgogne Franche-Comté/AgroSup Dijon, 1 esplanade Erasme, 21000 Dijon, France; 2 Département de Chimie et de Biochimie, Faculté des Sciences II, Université Libanaise, Jdeideth El Matn, Fanar 90656, Lebanon; 3 Département des Sciences de la Vie et de la Terre - Nutrition, Faculté des Sciences II, Université Libanaise, Jdeideth El Matn, Fanar 90656, Lebanon; 4 Institut de Chimie Moléculaire de l’Université de Bourgogne, UMR 5260, CNRS-12 Université de Bourgogne, 9 Avenue Alain Savary, 21078 Dijon, France

**Keywords:** copper (II) sensor, fluorescent chemosensor, tetraazamacrocycle rhodamine-based, water-soluble, acidic pH

## Abstract

For efficiently measuring copper (II) ions in the acidic media of white wine, a new chemosensor based on rhodamine B coupled to a tetraazamacrocyclic ring (13aneN_4_CH_2_NH_2_) was designed and synthesized by a one-pot reaction using ethanol as a green solvent. The obtained chemosensor was characterized via NMR, UV and fluorescent spectra. It was marked with no color emission under neutral pH conditions, with a pink color emission under acidic conditions, and a magenta color emission under acidic conditions where copper (II) ions were present. The sensitivity towards copper (II) ions was tested and verified over Ca^2+^, Ag^+^, Zn^2+^, Mg^2+^, Co^2+^, Ni^2+^, Fe^2+^, Pb^2+^, Cd^2+^, Fe^3+^, and Mn^2+^, with a detection limit of 4.38 × 10^−8^ M in the fluorescence spectrum.

## 1. Introduction

Copper is the third-most commonly found element on Earth and is one of the most extensively studied trace elements. It is vital for biological processes (e.g., energy generation, oxygen transport, signal transduction, detoxification, and blood clot formation) [1]. Copper also plays an important role in agriculture, where it was discovered to act as an inhibitor of fungal development when added to the soaking media of grains. Subsequently, its usage in agriculture gained momentum as copper is a vital component in the control of Bunt, a widely known seed-borne disease. Furthermore, copper compounds are applied in wine production as fungicides and bactericides [2]. These compounds—known as “Bordeaux mixture” (copper sulphate and lime in water) or “Burgundy mixture” (copper sulphate and sodium carbonate)—have for over a century been widely used in organic vineyards and farming [3]. Nowadays, thousands of tons are applied worldwide each year to prevent plant disease. The wine industry has become one of the leading sectors in agriculture in Europe (accounting for 60% of world production), followed by the United States, Australia, China, and South Africa [4]. Despite its importance in wine production, copper can lead to a deterioration of the quality of wine by either the formation of volatile sulfur compounds, or turbidity formation, or oxidation [5]. Therefore, it is of great importance to be able to carefully monitor and control the copper content in wine samples. One major drawback of copper control in wine is that the actual testing process requires complex and expensive equipment; an example is the flame atomic absorption spectroscopy, among others [6]. To improve the testing method and facilitate its implementation, analyzing copper content at the early stages of wine formation would be faster, easier, and cost-effective. In today’s science, probes have become the latest tool for practical detection of metal ions. They have a non-destructive nature, may allow ‘naked-eye’ detection, and require simple instrumentation, as well as being portable and easy to operate [7,8,9,10,11,12]. There has been a great improvement in copper detection in biological analysis thanks to the development of time- and cost-effective probes specific for copper detection at physiological pH [13,14,15,16,17]. The caveat is that, despite their appreciable implications, they are unsuited for usage in acidic media such as wine [18]. The ones based on rhodamine have attracted much attention for their excellent photophysical characteristics such as large absorption coefficient rate, high fluorescence quantum yield, and great photostability [19,20,21,22,23]. However, most rhodamine-based probes have limitations in practical usage, requiring complex multi-step operations using organic solvents which could create a toxic environment, as well as having a low water solubility [13,14,16,17,24,25]. Therefore, the principal aim of this research project was to develop a rhodamine-based probe by a one-pot synthesis for copper (II) ion detection in the acidic media of wine. One of the best ligand candidates for such a working scheme that was never coupled before to rhodamine B is a tetraazamacrocycle C-functionalized by an amino-methyl pendant arm. This macrocycle (**1**,13aneN_4_CH_2_NH_2_) has a high affinity towards copper (II) ions [26]. Moreover, it can overcome the above-mentioned limitations; namely, low water solubility, cross-sensitivities, multiple-step synthesis, and the need for co-solvents [8,27,28,29,30,31,32]. Additionally, and like most of the tetraazamacrocycles, it has a high water solubility where it can totally dissolve independently [33,34]. Interestingly, it has the ability to form stable complexes with bimetallic cations [26].

Consequently, based on the findings of this research study, we designed a rhodamine-based probe (Probe 3) specific for copper detection at acidic pH that is highly soluble in water, synthesized with a ‘one-pot’ synthesis reaction, and performs under green conditions. To the best of our knowledge, this is the first probe based on rhodamine coupled to a C-functionalized tetraazamacrocycle, and the first probe for direct copper detection in acidic pH of the white wine samples. The sensing characteristics were examined using a series of metallic cations where only Cu^2+^ ions were discriminated from the others.

## 2. Materials and Methods

### 2.1. Materials

The macrocycle 1 was purchased from Chematech S.A. (2 Rue Pauline Kergomard, 21000 Dijon, France). Rhodamine B, metallic salts and solvents were bought from Sigma-Aldrich (Saint-Quentin Fallavier, France) and used as received. All the solvents were of analytic grade. The salts used in stock solutions of metal ions were AgNO_3_, CaSO_4_, 3CdSO_4_.8H_2_O, CuCl_2_, FeSO_4_.7H_2_O, FeCl_3_.6H_2_O, MgSO_4_, Pb(CH_3_COO)_2_.3H_2_O, ZnCl_2_, Co(NO_3_)_2_.4H_2_O, NiCl_2_.H_2_O, and MnCl_2_.4H_2_O.

### 2.2. Instruments

#### 2.2.1. NMR Measurements

^1^H and ^13^C NMR spectra were recorded at the Plateforme d’Analyses Chimiques et de Synthèse Moléculaire de l’Université de Bourgogne on a Bruker 300 NMR spectrometer. Chemical shifts were reported in parts per million (ppm) using tetramethylsilane (TMS) as the internal standard. The following abbreviations are used; s: singlet, d: doublet, t: triplet, q: quartet, m: multiplet, br: broad.

#### 2.2.2. Mass Spectroscopy 

The high resolution and accurate mass measurements were carried out using a Bruker microTOF-Q™ ESI-TOF (electrospray ionization–time-of-flight) and a Thermo Scientific* LTQ Orbitrap mass spectrometer. 

#### 2.2.3. UV-Visible and Fluorescence Measurement

UV-visible absorption spectra were taken on a SAFAS-Monaco spectrophotometer using cuvettes STD UV Grade (280–800 nm) with a path length of 1 cm. Fluorescence emission spectra were carried out at room temperature using a Fluoromax-4 spectrofluorometer (Horiba Scientific, France) equipped with a xenon lamp. A 5-nm-wide slit was employed for both excitation (562 nm) and emission spectra (570–800 nm).

### 2.3. Synthesis and Characterization of Probe 3 [N-(9-(2-((1,4,7,10-tetraazacyclotridecan-5-yl)methyl)-3-oxoisoindolin-1-yl)-6-(diethylamino)-3H-xanthen-3-ylidene)- N ethylethanamine]

To a stirred solution of macrocycle (**1**) (0.125 g, 1.15 mmol, 50 mL of ethanol), rhodamine B (**2**) (0.11 g, 0.23 mmol) was added at room temperature. The reaction mixture was refluxed until the observation of the color alteration from dark pink to light orange (5 days). After cooling at room temperature, the solvent was removed and the residual orange oil was purified by flash chromatography (water:acetonitrile, 80:20). Filtrates were collected and the solvent was removed under vacuum and by lyophilization. Probe 3 was obtained as a pink powder (m = 121.8 mg, 0.19 mmol, yield: 40.9%). ^1^H NMR (300 MHz, D2O, 300 K) δ (ppm): 1.06 (t, 12H, J = 7 Hz), 1.77 (br s, 2H), 2.42–3.30 (m, 18H), 3.58 (m, 8H), 6.95 (d, 1H, J = 2.4 Hz), 6.98 (d, 1H, J = 2.4Hz), 7.12 (m, 3H), 7.43 (d, 1H, J = 2.2Hz), 7.52 (d, 1H, J = 2.2Hz), 7.61 (m, 2H), 7.90 (m, 1H), ^13^C{^1^H} NMR (75 MHz, D2O, 300 K) δ (ppm): 9.6 (× 2), 9.7 (× 2), 23.8, 41.8, 44.2, 45.2, 46.2, 47.2, 53.2 (× 2), 53.6 (× 2), 64.9, 111.5, 112.2, 114.4, 117.9, 118.2, 119.1, 119.7, 123.4, 124.0, 128.6, 130.1, 130.9, 134.8, 138.9, 139.3, 139.5, 151.9, 152.1, 152.2, 162.5, 163.0, 163.5, 171.8. HRMS (ESI): m/z calculated 641.4417 [M + H]^+^, found 641.438 [M + H]^+^. FT-IR: 1680 (C=O stretch), 1465 (Ar–C=C stretch), 1198 (C–N stretch), 1129 (C–O stretch) cm^–1^.

## 3. Results and Discussion

### 3.1. Preparation of Probe 3

**Scheme 1:**
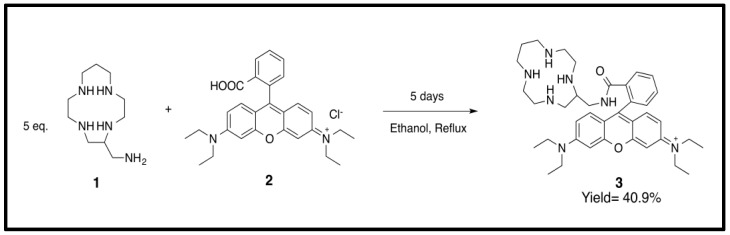
‘One-pot’ synthesis route of Probe 3.

The straightforward synthetic route used for designing Probe 3 is described in Scheme 1. Compound **1** was connected directly by its primary amine function of the tetraazamacrocyclic ring to the carboxylate group of Rhodamine B in reflux of ethanol (Scheme 1) and provided Probe 3 with a 40.9% yield after purification.

### 3.2. General Procedure for the Measurement of Absorption and Fluorescence Analysis

Probe 3 was dissolved in distilled water to make a 2 × 10^−3^ M stock solution. It was diluted further depending on the concentration needed for measurement. As for copper, a 2 × 10^–3^ M of copper (II) ions solution was prepared by dissolving CuCl_2_ in distilled water. It was diluted further depending on the concentration needed for measurement. First of all, we tested the behavior of Probe 3 at different pH values alone as well as in the presence of an equimolar content (1 × 10^–4^ M) of copper (II) ions. Two sets of cuvettes of 2 mL were prepared. In the first set, 1 mL of Probe 3 (2 × 10^–4^ M) solution and 1 mL of each buffer solution were added to the cuvettes of 2 mL to test the behavior of Probe 3 alone at different pH conditions. In the second set, 1 mL of Probe 3 (2 × 10^–4^ M) solution and 1 mL of copper (II) ions (2 × 10^–4^ M) diluted in the various buffer solutions were added to the cuvettes of 2 mL to test the behavior of Probe 3 in the presence of copper (II) ions at different pH conditions. Then, we studied the fluorescence spectra of Probe 3 (1 µM) upon adding increased concentrations of copper (II) ions (0–1 µM) at the same acidic pH (pH 4.7). Cuvettes of 2 mL were prepared as follows: 1 mL of Probe 3 (2 µM); varying volumes (between 0–1 mL) of copper (II) ions solution (2 µM); and the buffer solution (pH 4.7; 0.1 M potassium dihydrogen phthalate buffer solution 10/2.72, v/v) to the full scale. Finally, we tested the metal-ion selectivity of Probe 3 (1 µM) in acid conditions (pH 4.7) towards copper (II) ions at an equimolar ratio with copper (II) ions and the other competitor metal ions (1 µM). All the cuvettes were vigorously mixed. The absorption and the fluorescence sensing of Cu^2+^ ions were run directly after the mixing since the color change from pink to magenta took place immediately.

### 3.3. The pH Dependency of Probe 3 and Its Behavior Towards the Absence or Presence Cu^2+^ Ions Explained by UV-Vis Measurement

**Figure 1:**
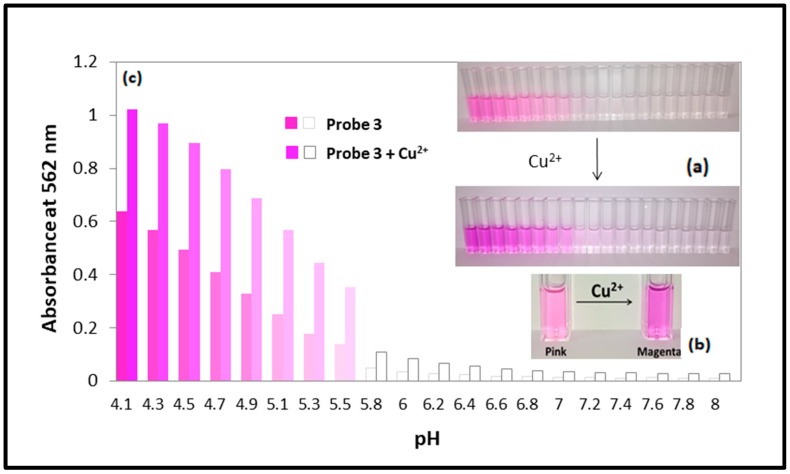
(**a**) Inset images showing the color change of 1 × 10^–4^ M Probe 3 alone (upper image) and in the presence of 1 × 10^–4^ M copper (lower image) at various pH conditions ranging from 4.1 to 8 (left to right in both), as well as the color intensity variation with changing the pH conditions. (**b**) Inset image showing the intense magenta color development upon addition of 1 × 10^−4^ M Cu^2+^ ions to a solution of 1 × 10^–4^ M Probe 3 in an acidic pH environment (4.7). (**c**) UV-visible spectra at 562 nm of 1 × 10^–4^ M Probe 3 at different pH values (4.1 to 8) alone and in presence of 1 × 10^–4^ M Cu^2+^ ions.

The affinity of Probe 3 (1 × 10^–4^ M) and its response to copper (II) ions addition at an equimolar ratio (1 × 10^–4^ M) was firstly investigated at different pH conditions (buffer solutions: 4.1; 4.3; 4.5; 4.7; 5.1; 5.3; 5.5; 5.8; 6; 6.2; 6.4; 6.6; 6.8; 7; 7.2; 7.4; 7.6; 7.8; 8). The color and intensity change upon addition of copper (1 equiv.) at different pH values were observed and measured by the spectrophotometer (Figure 1).

**Scheme 2:**
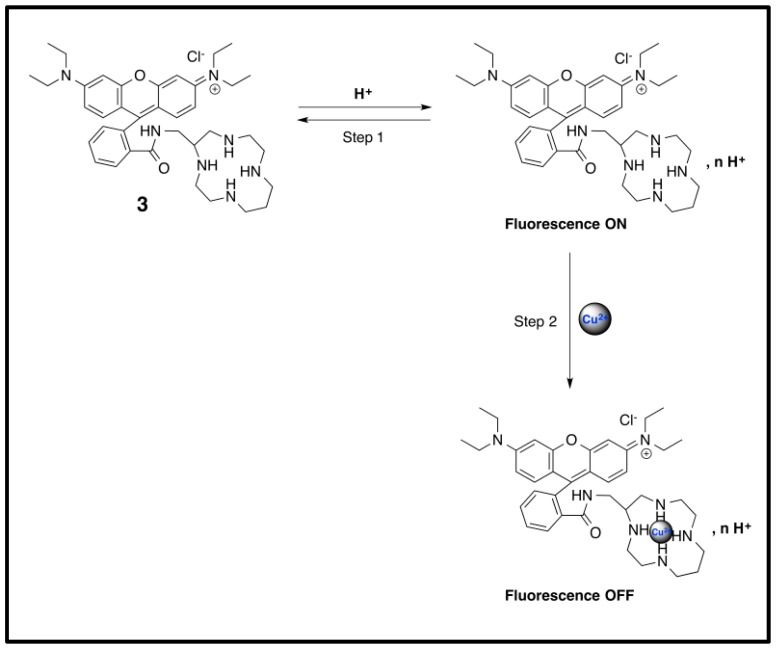
The proposed reaction mechanism of 1 × 10^–4^ M Probe 3 alone in acidic pH (Step 1: protonation of the macrocyclic part and development of the naked-eye-detected pink color) and upon addition of 1 × 10^–4^ M copper (II) ions (Step 2: color change from pink to the naked-eye-detected magenta color upon addition of copper (II) ions).

Figure 1 illustrates a clear ‘naked-eye’-detected color change of Probe 3 solutions from pink to magenta upon addition of copper (II) ions. Moreover, it shows a change in the color intensity that is inversely proportional to the pH values in all samples. At acidic pH, the protons induce the protonation of the macrocyclic part which induces consequently the development of the pink color which is detected by the ‘naked eye’. The development of the pink color is dependent on the acidic pH. Upon addition of the copper (II) ions to the acid media containing Probe 3, the pink color changes to magenta. The development of a noticeable ‘naked-eye’-detected magenta color of the probe upon metal addition can be explained by a metal complexation of Cu^2+^ ions in the macrocyclic ring [26] (Scheme 2).

### 3.4. Fluorescence Measurements of Probe 3 in the Presence of Copper (II) Ions at Different Concentrations

**Figure 2:**
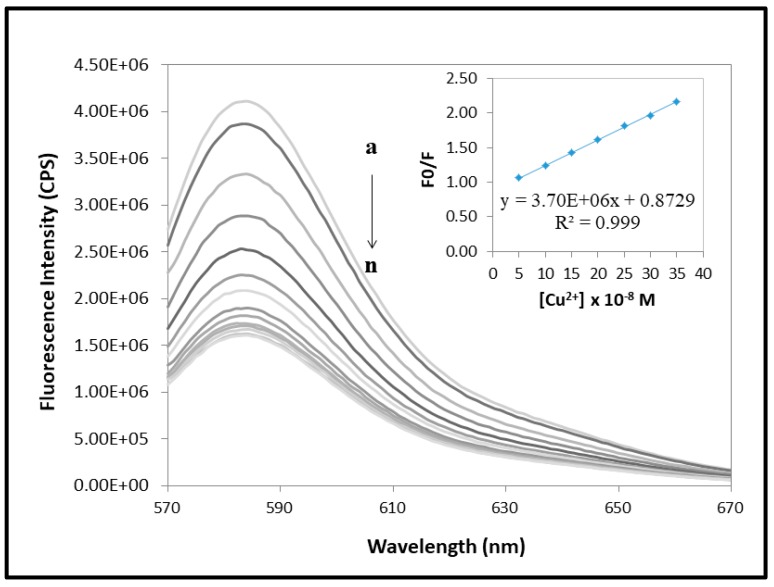
Fluorescence titrations (λ_ex_: 583 nm) of Probe 3 (1 µM) in acid conditions (pH 4.7) with addition of [Cu^2+^] a: 0 M, b: 5 × 10^–8^ M, c: 1.0 × 10^–7^ M, d: 1.5 × 10^–7^ M, e: 2.0 × 10^–7^ M, f: 2.5 × 10^–7^ M, g: 3.0 × 10^–7^ M, h: 3.5 × 10^–7^ M, i: 4.5 × 10^–7^ M, j: 5.0 × 10^–7^ M, k: 6.5 × 10^–7^ M, l: 7.0 × 10^–7^ M, m: 8.5 × 10^–7^ M, n: 1 µM.

Emission titration experiments were conducted in order to understand the sensing nature of Probe 3 in acidic media (pH 4.7) with the progressive addition of Cu^2+^ ions at low concentrations undetected by the UV-Vis spectra. Since metal ions are usually found in trace quantities, it is imperative to study the behavior of our probe at lower concentrations. Spectral analyses were investigated between 10^–8^ M and 1 µM with 10^–8^ M being the lowest detection limit. According to the European Food Safety Authority food composition database, copper content in French food is higher (around 3.5 × 10^–4^ mol/100 g) than in European agricultural soils (between 2.5 × 10^–4^ mol/kg and 9 × 10^–4^ mol/kg) [35,36]. Hence, such data indicate that the detection range of Probe 3 is adapted for copper analysis in both food and soil. The reaction of Probe 3 with copper (II) ions produced clear fluorescence emission changes (Figure 2).

The excitation wavelength was at 562 nm. While absorbance intensities increased upon gradually adding copper (II) to Probe 3, emission intensities decreased. The response of Probe 3 indicates no band shift in the UV-Vis and fluorescence spectra, unlike other rhodamine-based probes reacting with metal ions [23,37]. In our case, there is an increment of the absorbance (Figure S5) and a decline in the intensity of emission bands centered at the same wavelength. Both changes implicate the formation of a metal complex with Probe 3. These effects could explain the quenching activity of copper, which decreases the intensity value of the emission band of Probe 3 alone centered at 583 nm by 2.5-fold upon addition of an equivalent quantity of copper. The fluorescence emission spectrum is affected with an energy-transfer mechanism for quenching. The quenching interaction appears as the copper(II)–macrocycle complex is formed near the Rhodamine B [38,39,40]. 

### 3.5. Limit of Detection (LOD) of Copper (II) Ions by Probe 3

According to Figure 2, the results show that the fluorescence intensity of Probe 3 significantly decreased with increasing copper (II) ion concentration [Cu^2+^]. The relationship between F0/F and the concentration of copper (II) ions from 5 × 10^–8^ to 3.5 × 10^–7^ M can be described by a linear equation (correlation coefficient: 0.9990) as follows: F0/F = 3.70 × 10^6^ × [Cu^2+^] + 0.8729. The signal-to-noise ratio (S/N) is equal to 3. When the signal-to-noise ratio is equal to three, it is generally accepted for estimating LOD [41]. The LOD is calculated by multiplying the standard deviation of the y-intercepts of regression lines by 3 and then dividing the obtained value by the slope of the calibration curve [41,42]. The LOD for the detection of copper (II) ions obtained is 4.38 × 10^–8^ M. This result indicates that Probe 3 is highly sensitive to the detection of copper (II) ions.

### 3.6. Selectivity of Probe 3 Toward Cu^2+^ Ions over Other Metal Ions

**Figure 3:**
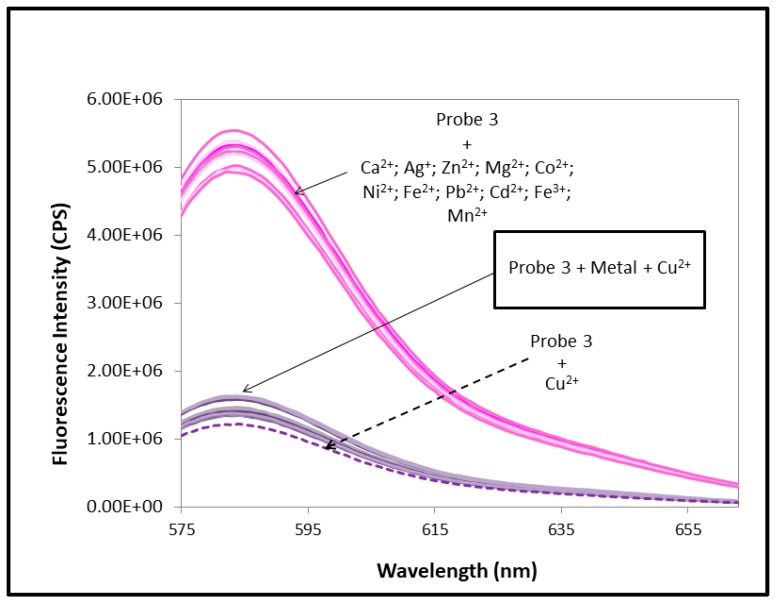
Metal-ion selectivity of Probe 3 (1 µM) in acid conditions (pH 4.7). The pinkish lines represent the emission of the 11 solutions containing Probe 3 (1 µM) and 1 equiv. of one of the following metal ions: Ca^2+^; Ag^+^; Zn^2+^; Mg^2+^; Co^2+^; Ni^2+^; Fe^2+^; Pb^2+^; Cd^2+^; Fe^3+^; and Mn^2+^. The magenta lines represent the fluorescence quenching of a solution of Probe 3 (1 µM) and 1 equiv. of the cations of interest upon addition of 1 equiv. of copper (II) cations. The magenta pointed line represents the fluorescence emission of a solution of Probe 3 (1 µM) and 1 equiv. of copper (II) cations.

One basic characteristic of evaluating the effectiveness of a chromophore is investigating its selectivity toward a targeted metal ion in the presence of other metal ions at low concentrations. Therefore, the fluorescence of Probe 3 (1 µM) in the presence of other metal ions (1 µM each), including Ca^2+^, Ag^+^, Zn^2+^, Mg^2+^, Co^2+^, Ni^2+^, Fe^2+^, Pb^2+^, Cd^2+^, Fe^3+^, and Mn^2+^, was studied under the same conditions (Figure 3). 

As shown in Figure 3, it is clearly evident that the other metal ions did not generate any significant changes; the fluorescence intensity of Probe 3 alone and upon addition of these competitive metal ions did not vary significantly, unlike the results obtained after addition of copper (quenching of fluorescence). For further confirmation of the selectivity of Probe 3 towards Cu^2+^ ions, a competitive experiment between copper and the other metals was performed under the same conditions. Figure 3 shows a quenching of fluorescence upon addition of copper to the samples containing the metal ions and Probe 3. The results indicate a distinct selectivity and affinity of Probe 3 for copper even in the presence of other metal ions. This might prove the high suitability of Probe 3 for copper (II) ion detection in terms of geometry conformation, electron configuration, size, and charge. Therefore, Probe 3 could be considered a reliable copper (II) ion detector, independently from the presence or absence of other metal ions (Ca^2+^, Ag^+^, Zn^2+^, Mg^2+^, Co^2+^, Ni^2+^, Fe^2+^, Pb^2+^, Cd^2+^, Fe^3+^, and Mn^2+^).

### 3.7. Application Test of Probe 3 in White Wine Samples

Increasing attention has been paid by wine producers to the improvement of winemaking [43]. Copper (II) ion content is one of those elements that should be controlled for improving wine quality and preventing oxidative spoilage, color deterioration, and turbidity formation [6,44,45]. The copper content at the early stages of wine formation influences the quality of the subsequent wine. Elevated concentrations can cause oxidative spoilage, leading to red wine attaining a pink hue and white wine going brownish, as well as to haze formation. The generally recommended ‘safe’ copper concentration (total copper) in wine is below 0.3–0.5 mg/L (i.e., 4.7 × 10^–6^ M to 7.9 × 10^–6^ M) [6]. We wanted to test the concentration of copper in a Burgundy Chardonnay of 2016. Six samples of the white wine (WS1, WS2, WS3, WS4, WS5, and WS6) buffered at pH 4.7 were tested in triplicate for copper (II) ions content. They also were compared to the following controls: control 1 containing Probe 3 alone at the same pH conditions; and control 2 containing a wine model without copper. The results are shown in Table 1. The results of this study show that, for white wine samples, the specifically-designed chemosensor Probe 3 is able to detect traces of copper (II) ions at low concentrations and in acidic conditions (pH 4.7). The concentration of copper (II) ions sought in each of these samples was between 0 and 1 mg/L. Wine samples 1 and 2 are therefore safer than the other samples (WS3, WS4, WS5, and WS6) as they contained less than 0.3–0.5 mg/L copper (II) ions.

## 4. Conclusions

The aim of this study was to design a highly efficient fluorescent chemosensor with a high selectivity toward copper (II) ions over other competitive metals (Ca^2+^, Ag^+^, Zn^2+^, Mg^2+^, Co^2+^, Ni^2+^, Fe^2+^, Pb^2+^, Cd^2+^, Fe^3+^, and Mn^2+^) in acidic media with a limit of detection equal to 4.38 × 10^–8^ M. To the best of our knowledge, Probe 3 is the first chemosensor based on rhodamine B (**2**) and a tetraazamacrocycle C-functionalized on the aminomethyl pendant arm (**1**). It is prepared in a ‘one-pot’ reaction and under green conditions. It is highly soluble in water and has a high acidic pH tolerance. We believe it has the potential of becoming a promising tool for rapid and environmentally-friendly copper (II) ion detection and control for the wine industry. The acidic pH permits the formation of the naked-eye-detected pink color by the protonation of the macrocycle of Probe 3. The addition of copper (II) ions leads to the formation of the copper (II)–macrocycle complex responsible for the remarkable fluorescence quenching of the rhodamine B of Probe 3 accompanied by a naked-eye-detected color change from pink to magenta. Probe 3, thus, may provide a most efficient access to a large range of usages in complex acidic food matrices for copper (II) ion detection and control.

## Figures and Tables

**Table 1:** Results of copper (II) ions present in white wine samples with different copper concentrations.

Sample	WS1	WS2	WS3	WS4	WS5	WS6	Controls
Cu^2+^ content (mg/L) ^1^	0.03 ± 0.01	0.15 ± 0.01	0.38 ± 0.01	0.44 ± 0.02	0.68 ± 0.00	0.76 ± 0.00	0.00 ± 0.00

^1^ Mean of three replicates (±SD).

## Supplementary Materials

The following are available online at https://www.mdpi.com/1424-8220/19/20/4514/s1.
