# Supplementary material for: A New pH-Dependent Macrocyclic Rhodamine B-Based Fluorescent Probe for Copper Detection in White Wine
Source: Sensors (Basel). 2019 Oct 17;19(20):4514. doi: 10.3390/s19204514 (PMC6832540; doi:10.3390/s19204514)
Supplement: Supplementary file 1 [file sensors-19-04514-s001.pdf]

Supplementary Materials

A new pH dependent macrocyclic Rhodamine B-based fluorescent probe for copper detection in white wine

Nour Doumani, Elias Bou-Maroun, Jacqueline Maalouly, Maya Tueni, Adrien Dubois, Claire Bernhard, Franck Denat, Philippe Cayot and Nicolas Sok*

Table of contents

NMR measurements......2

^{1}H and ^{13}C NMR spectra were recorded on a Bruker 300 and 500 NMR spectrometer......2

Figure 1: ^{1}H NMR spectrum Probe 3 (300 MHz, D2O, 300 K)......2

Figure 2: ^{13}C NMR of probe 3 (75 MHz, D2O, 300 K)......2

HR-mass measurements......3

Figure 3: Figure 3: ESI-TOF spectrum of Probe 3......3

FT-IR measurements......4

Figure 4: FTIR spectrum of Probe 3......4

UV-vis spectra of Probe 3......5

Figure 5: UV-visible absorption spectra of Probe 3 (1 μM) in buffer solution 4.7 with addition of [Cu^{2+}]......5

Figure 6: Regression curve of the absorbance of Probe 3 alone at different concentrations in distilled water.. 5

NMR measurements

^{1}H and ^{13}C NMR spectra were recorded on a Bruker 300 and 500 NMR spectrometer. Chemical shifts were reported in parts per million using tetramethylsilane (TMS) as the internal standard.

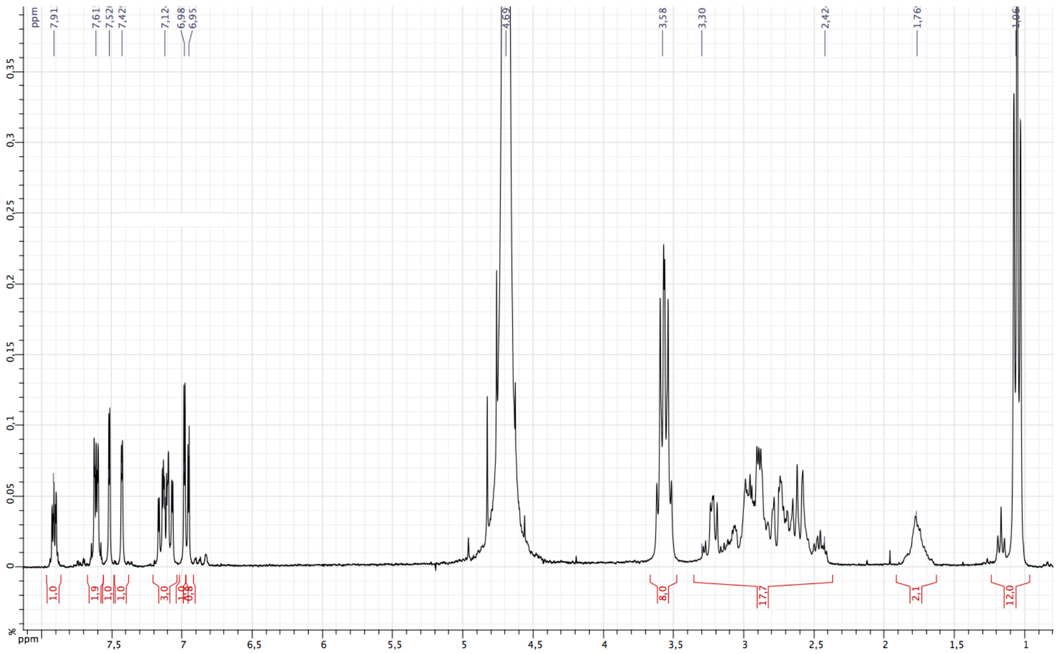

Figure 1. ^{1}H NMR spectrum Probe 3 (300 MHz, D2O, 300 K).

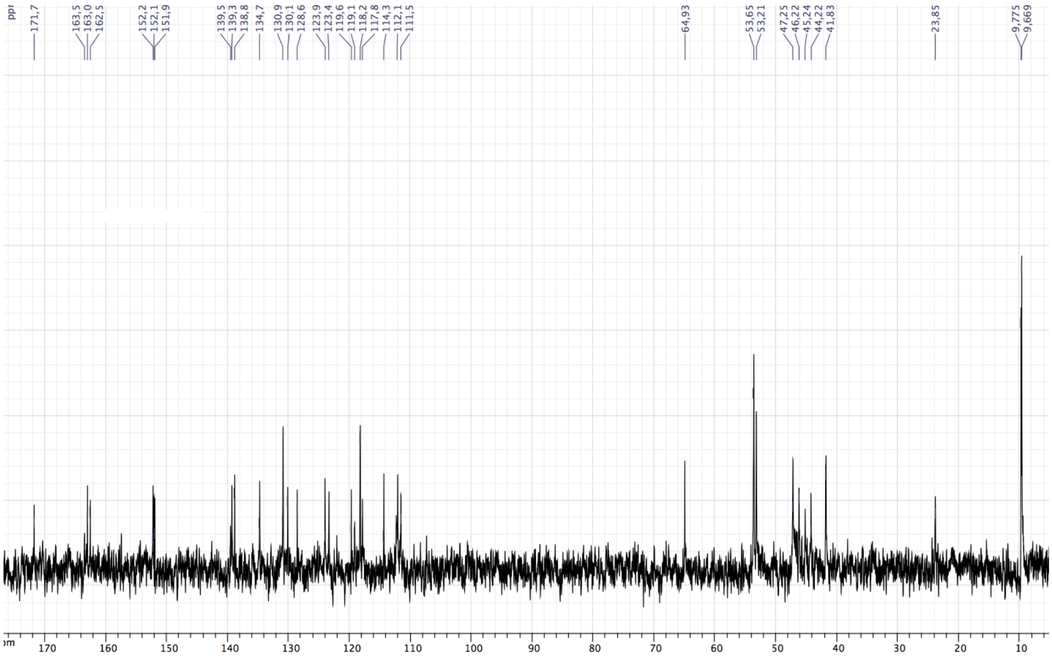

Figure 2. ^{13}C NMR of Probe 3 (75 MHz, D_2O, 300 K).

HR-mass measurements

The high resolution and accurate mass measurements were carried out using a Bruker microTOF-Q™ ESI-TOF (Electro Spray Ionization – Time of Flight) and a Thermo Scientific* LTQ Orbitrap mass spectrometer.

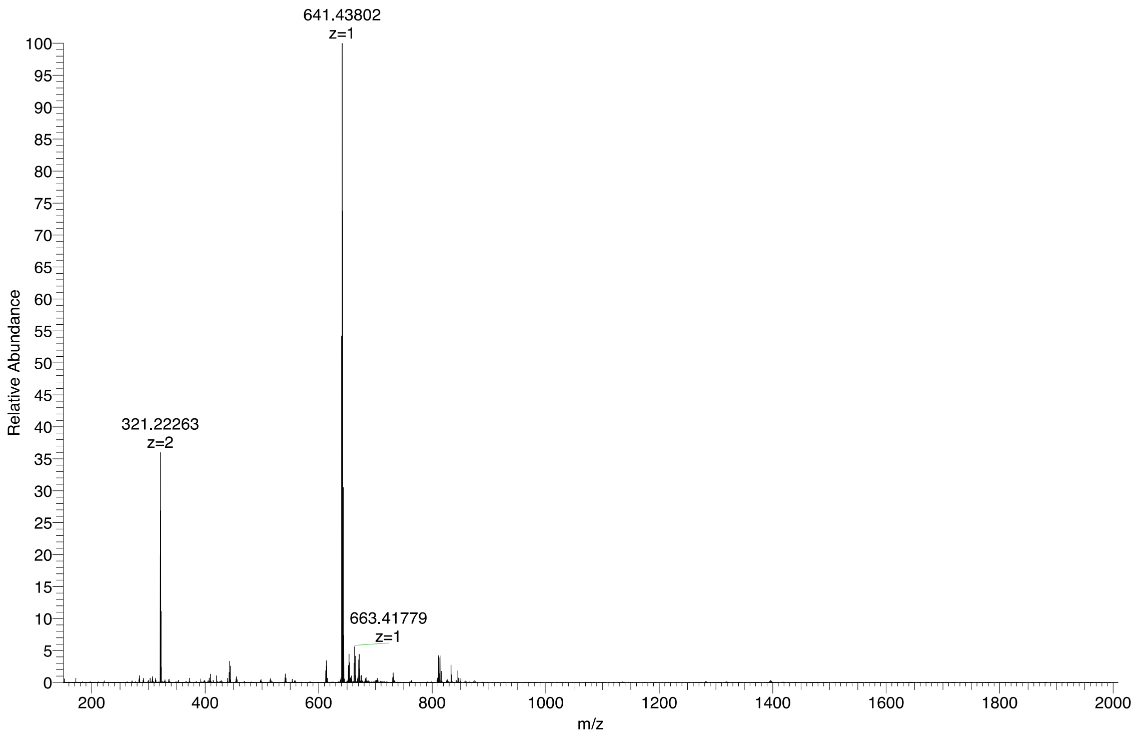

Figure 3. ESI-TOF spectrum of Probe 3.

FT-IR measurements

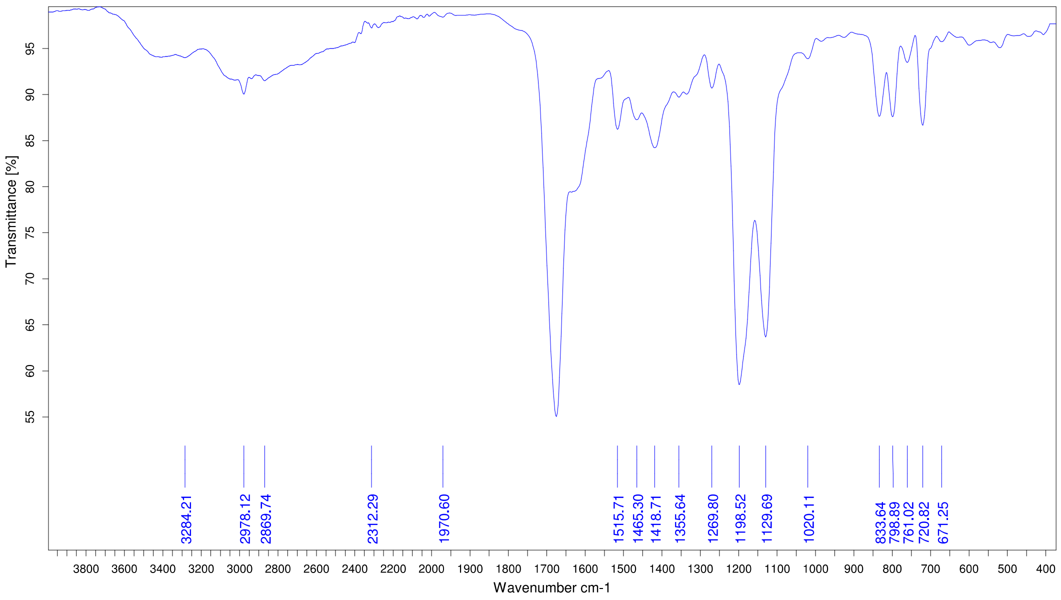

Figure 4. FTIR spectrum of Probe 3.

UV-vis spectra of Probe 3 in presence of copper (II) ions at different concentrations

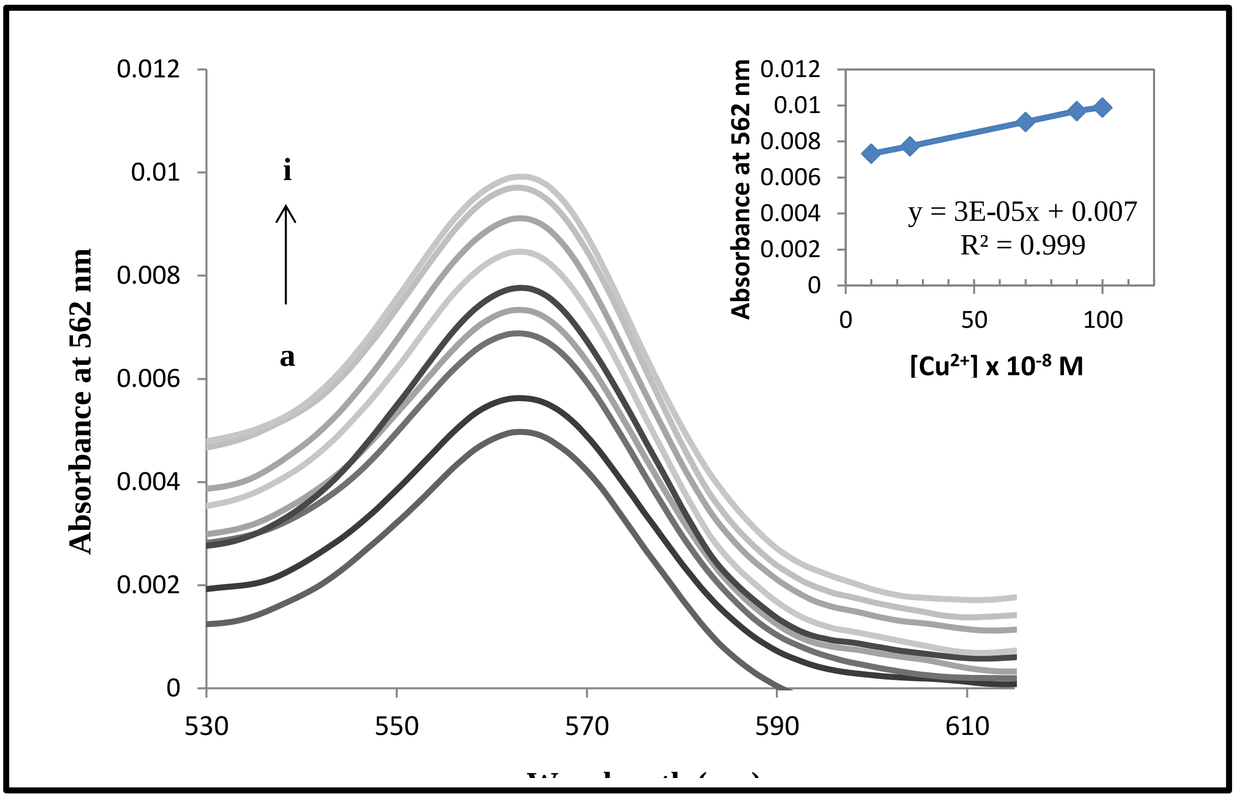

Figure 5. UV-visible absorption spectra of Probe 3 (1 μM) in buffer solution 4.7 (0.1 M potassium dihydrogen phthalate buffer solution 10/2.72, v/v) with addition of [Cu^{2+}] a: 0 M, b: 5 × 10^{-8} M, c: 10 × 10^{-8} M, d: 25 × 10^{-8} M, e: 35 × 10^{-8} M, f: 45 × 10^{-8} M, g: 70 × 10^{-8} M, h: 90 × 10^{-8} M, i: 1μM.

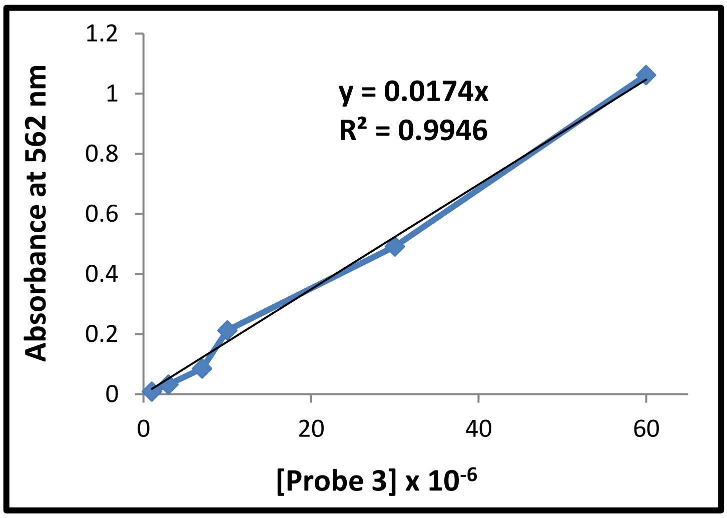

Figure 6. Regression curve of the absorbance of Probe 3 alone at different concentrations (from 0 to 60·10^{-6} M) in distilled water.

ε = 0.0174 L/mol × cm

$\varepsilon = \frac{A}{Cl}$; A: absorbance = y; C: concentration = x (mol/L); l: width of cuvette = 1 (cm).
